# Supplementary material for: Variabilities and contentions in anesthesiologists’ perspectives on Japanese perianesthesia nurses: A qualitative study
Source: PLoS One. 2024 Dec 31;19(12):e0313158. doi: 10.1371/journal.pone.0313158 (PMC11687901; doi:10.1371/journal.pone.0313158)
Supplement: S1 Appendix — (DOCX) [file pone.0313158.s001.docx]

**S1 Appendix: Interview guide**

**First question**

Could you please outline the primary responsibilities of perianesthesia nurses in your hospital?

**Follow-up questions**

On a scale of 0 to 10, how satisfied are you with your perianesthesia nurses' job?

Please tell us why you gave it that score.

**Additional questions**

Can you tell us about a particularly memorable episode regarding perianesthesia nurses?

What challenges did you encounter?

How did you address/respond to them?

In your opinion, what qualities define an ideal perianesthesia nurse?

(If the interviewee is the person who decided on the PAN duties)

　 Why did you decide to delegate that task to PAN?

Why did you decide not to delegate that task to PAN?
