# Supplementary material for: Variabilities and contentions in anesthesiologists’ perspectives on Japanese perianesthesia nurses: A qualitative study
Source: PLoS One. 2024 Dec 31;19(12):e0313158. doi: 10.1371/journal.pone.0313158 (PMC11687901; doi:10.1371/journal.pone.0313158)
Supplement: S2 Appendix — (DOCX) [file pone.0313158.s002.docx]

**S2 Appendix: Final analytical framework**

**I. Anesthesiologists' perspectives on the implementation of the PAN system in Japan**

1. Shortage of anesthesiologists

2. Effective interprofessional collaboration and task shifting

3. The emergence of freelance anesthesiologists with lower qualifications and higher remuneration

4. Prevention of surgeon-led establishment of anesthesia nurse system

< MEMO >

• There are a variety of social factors

• Causes of the anesthesiologist shortage:

- Absolute / Relative shortage

- Increase in surgical case volume

- Rise in complex surgeries

- Expansion of anesthesia practice areas

• Keywords indicating relations among themes:

- Increased variety and volume of anesthesia work / Increased need for PAN

- Unnecessary anesthesiologists

- A reluctance toward collaboration between PANs and surgeons

**II. High appraisal of current PANs**

1. Outstanding qualities

2. High-level tasks

< MEMO >

• Both the inner nature and the actual work are excellent

• For example:

- High motivation

- Interpersonal competence (influenced by graduate studies)

- Successful anesthesia work with PANs

- Contribution to advanced collaborative care

• Keywords indicating relations among themes:

- Anesthesiologists are satisfied

- Current PANs are ideal

- The opposite of ideal is inadequate

- Commonalities with exemplary PANs

**III. Anesthesiologists' expectations**

1. Expectations for PANs

2. Benefits of the PAN system

3. Exemplary PANs

< MEMO >

• Expect to assist anesthesiologists: less workload on anesthesiologists

• Take advantage of nurses' unique abilities: Improve safety, quality of care, and patient satisfaction

• Narrative about the ideal perianesthesia nurse

• Keywords indicating relations among themes:

- Anesthesiologists are satisfied with the PAN and PAN system

- PAN Specialty / Difference between anesthesiologists and PANs

- Expectations differ among anesthesiologists: Administering anesthesia and/or other tasks.

**IV. Anesthesiologists' perspectives on PAN anesthesia**

1. Conflicting views on PAN anesthesia

2. The Anesthesiologists' concerns in PAN anesthesia

3. Essentials for safe PAN anesthesia

< MEMO >

• 2 conflicts in category 1

**Conflict 1**

- Attitudes toward PAN anesthesia: "Support" or "NOT Support"
- Two types of "NOT support": "Not actively in favor" and "explicitly oppose"

**Conflict 2**

- Substituting anesthesiologists with PANs: "Agree" or "Disagree"

• Despite varying opinions in Conflict 1, numerous anesthesiologists have raised diverse concerns about PAN anesthesia

• Safety mechanisms are being proposed, including initiatives by anesthesiologists themselves and organizational policies.

• Keywords indicating relationships among themes:

- Patient's feelings of rejection of PANs

- Uncertainty about laws and related matters

**V. Anesthesiologists' perspectives on the PAN system**

1. Challenges in the operation of PANs

2. Recommendations for the advancement of the PAN system

3. Participation of stakeholders

< MEMO >

• Various problems in the system were identified through their interactions with PANs

• Suggestions are proposed for enhancing the system for PANs, anesthesiologists, and Japan

• Anesthesiologists have identified perceived limitations in the system, leading to requests for the participation of public institutions:

- Japanese Society of Anesthesiologists

- Japanese Society for Perianesthesia Nursing and Medicine

- Nursing Association

- The Japanese government

• Keywords indicating relations among themes:

- Uncertainty about laws and related matters

- Enhancing the system in each hospital alone is challenging
