# Supplementary material for: Variabilities and contentions in anesthesiologists’ perspectives on Japanese perianesthesia nurses: A qualitative study
Source: PLoS One. 2024 Dec 31;19(12):e0313158. doi: 10.1371/journal.pone.0313158 (PMC11687901; doi:10.1371/journal.pone.0313158)
Supplement: S3 Appendix — (PDF) [file pone.0313158.s003.pdf]

Partcipant #6

157 A: Actually, nurses often earn less than anesthesiologists, even if they work as PANs.

158 It's possible that they might be seen as cheap labor.

159 I'm concerned about that happening.

> Theme IV:
> 1. Conflicting Views on PAN Anesthesia in Substituting PANs for Anesthesiologists - Disagree

160 For example, if the managements want to increase the number of surgeries performed or has plans to do so, they might think that hiring PANs at a lower cost is a solution.

161 However, this could lead to the hired PANs being treated as nothing more than "anesthesia machines".

162 This would be unfortunate for PANs.

> Theme V:
> 1. Challenges in the operation of PANs Addressing demotivating factors for PANs

163 Perioperative nurses are passionate about anesthesia, but they also want to provide a broader range of care and nursing services throughout the perioperative period, rather than just focusing on the number of cases.

164 I hope it doesn't turn out that way as the number of perioperative nurses increases.

165 So, I think it's crucial to have the recognition that an appropriate number of anesthesiologists is needed to match the increase in case volume.

> Theme IV:
> 2. The Anesthesiologists' concerns in PAN anesthesia Risks of the diminishing value of anesthesiologists

166 Q: Yes, I agree. It's a plausible line of thinking for management to consider hiring PANs instead of anesthesiologists because they're cheaper.

167 A: Exactly, I think so. It's definitely a possibility.

168 Q: So, I think there might be some concerns about this.

169 Have you had any discussions with other colleagues about how to prevent this or any similar situations?

170 A: We haven't delved into that yet.

171 In fact, most hospitals are not aware of the existence of PANs.

172 I'm concerned about what will happen in the future as perioperative nurses become more widespread.

173 Fortunately, currently PANs are mostly found in educational hospitals.

174 They are in university hospitals or hospitals like university settings.

175 There aren't many PANs outside of these educational hospital environments.

176 However, in the future, when they start appearing more in community hospitals, there might be people who think, "Why not hire them and let them administer anesthesia at our hospital too?"

177 I have some concerns about that happening in about ten years or so.

> Theme IV:
> 1. in Substituting PANs for Anesthesiologists - Disagree
> 2. The Anesthesiologists' Concerns in PAN Anesthesia Concerns Regarding the Use of PANs for Cost Reduction

178 Q: Do you think it can be prevented?

179 A: I'm not sure.

180 We'll have to assess what kind of managers, or rather, what kind of hospitals they are.

181 From the perspective of PANs, it might be a good idea to assess that.

182 But even so, if, for example, the salary is reasonably good, they might be inclined to go there.

> Theme IV:
> 3. Essentials for Safe PAN Anesthesia Establishment of PAN education system by anesthesiologists - Esteeming the philosophy of being a PAN

183 Of course, even if it's cheaper than hiring an anesthesiologist, if the pay is significantly higher than that of a regular nurse, it might still be appealing.

> Theme V:
> 2. Recommendations for the advancement of the PAN system. Clarification of Benefits for PANs

184 Q: In that case, hospitals might find it more cost-effective to hire someone who can handle things to a certain extent, increase the case volume, and boost revenue.

185 A: That's true.

186 Q: Consequently, the demand for anesthesiologists might decrease.

187 A: Regrettably, there's a possibility.

> Theme IV:
> 2. The Anesthesiologists' Concerns in PAN Anesthesia Risks of the diminishing value of anesthesiologists
