# Supplementary material for: Variabilities and contentions in anesthesiologists’ perspectives on Japanese perianesthesia nurses: A qualitative study
Source: PLoS One. 2024 Dec 31;19(12):e0313158. doi: 10.1371/journal.pone.0313158 (PMC11687901; doi:10.1371/journal.pone.0313158)
Supplement: S5 Appendix — (PDF) [file pone.0313158.s005.pdf]

This set of tables presents a comprehensive overview of the coding structure derived from our qualitative study on anesthesiologists' perspectives towards perianesthetic nurses. The table is organized into five main themes, each containing multiple categories and their associated codes with corresponding quotation data. Theme IV is further elaborated through three separate tables, each focusing on one of its three categories for more detailed analysis.
For each code, we have provided one or two representative quotations along with their reference numbers. Quotations marked with an asterisk (*) indicate those that have been used as illustrative examples in the main text of the manuscript.
Theme IV's second category was presented as Table 2 in the main text.

Table of contents

I. Anesthesiologists' perspectives on the implementation of the PAN system in Japan
1. Shortage of anesthesiologists
2. Effective interprofessional collaboration and task shifting
3. Emergence of freelance anesthesiologists with lower competency and higher remuneration
4. Prevention of surgeon-led establishment of an anesthesia nurse system

II. High appraisal of current PANs
1. Outstanding qualities
2. High-level tasks

III. Anesthesiologists' expectations
1. Expectations for PANs
2. Benefits of implementing the PAN system
3. Exemplary PANs

IV. Anesthesiologists' perspectives on PAN anesthesia
1. Conflicting views on PAN anesthesia
2. Anesthesiologists' concerns in PAN anesthesia
3. Essentials for safe PAN anesthesia

V. Anesthesiologists' perspectives on the PAN system
1. Challenges in the operation of PANs
2. Recommendations for the advancement of the PAN system
3. Participation of stakeholders

I. Anesthesiologists' perspectives on the implementation of the PAN system in Japan

	category	code	interview/ quote no.	quote summary
1	Shortage of anesthesiologists	Absolute shortage	3/q38, q100	There is a shortage of anesthesiologists. If PANs perform anesthesia, it can compensate for the lack of anesthesiologists[...]. This shortage is even more pronounced in rural areas. It is common for a single anesthesiologist to manage anesthesia for four cases simultaneously.
			9/q87*	Community hospitals face severe understaffing. The COVID-19 pandemic has exacerbated this issue, with many anesthesiologists being reassigned to ICU duties out of necessity. This situation has created a shortage of anesthesiologists available for operating room duties.
		Hectic daily schedules	12/q132	I am very busy every day. Freelancers are expensive, so we can't hire many. The staff is also exhausted.
		Rise in complex surgeries	16/q139	It's acceptable to let nurses handle anesthesia for ASA-PS1 and 2 patients. There are many surgeries that need highly skilled anesthesiologists, such as cardiac surgery, pediatric cardiac surgery, one-lung ventilation, and obstetric anesthesia.
		Expansion of anesthesia practice areas	6/q28	The relative shortage of anesthesiologists is due not only to the high volume of operating room duties but also to an increase in anesthesia-related tasks, such as pain-free deliveries and sedation outside the operating room. Each of these additional duties requires the involvement of an anesthesiologist.
2	Effective interprofessional collaboration and task shifting	Expectations for task shifting to nurses	13/q162	In Japan, Work Style Reform for hospital doctors is progressing. This aligns with the current societal trend. In this context, I believe it would be appropriate to delegate certain tasks to PANs where possible.
		Interprofessional collaboration	24/q18	Anesthesiologists alone are not sufficient. The involvement of nurses further enhances safety and quality. The differing perspectives of physicians and nurses are important. Only PANs can provide seamless care across outpatient surgical clinics, operating rooms, and postoperative wards.
			20/q30	There's a bit of a bias in anesthesiologists' capabilities and differences in their backgrounds. Including nurses in anesthesia care might provide a slightly different perspective and experience, potentially improving patient care and support.
3	Emergence of freelance anesthesiologists with lower competency and higher remuneration	Complaints about freelance anesthesiologists	16/q139	Freelance anesthesiologists pose a problem. Although some are highly skilled, others may not manage anesthesia properly, potentially leading to accidents. This has become a recognized issue, with concerns about paying high anesthesia fees to such practitioners.
			23/q223	Initially, the only available anesthesiologists were these nasty freelancers. This situation led to the idea that nurses might be a better alternative for anesthesia roles.
		Cost-effectiveness of PANs	9/q218	Freelance anesthesiologists are costly, while PANs, although not physicians, can perform anesthesia procedures flawlessly. If hospital administrators were asked to choose between these options, the preferable choice would be evident.
4	Prevention of surgeon-led establishment of an anesthesia nurse system	Anesthesiologists' leadership in involving nurses*	23/q228	In the past, some cardiac surgeons attempted to delegate anesthesia to nurses and dentists. If such practices are allowed, young doctors may be dissuaded from becoming anesthesiologists, which would not benefit the public. As nurses become involved, it is essential that anesthesiologists take a leading role.
		A reluctance toward collaboration between PANs and surgeons	12/q132	Hire only nurses and have them administer anesthesia under the surgeon's direction. It's risky.
			16/q163*	The registration of perianesthesia nurses should be managed by organizations such as the Japan Society of Anesthesiologists. It's essential to ensure that PANs are placed in settings where anesthesiologists are present. If they were to start administering anesthesia independently with surgeons, it could lead to serious problems.

II. High appraisal of current PANs

	category	code	interview/ quote no.	quote summary
1	Outstanding qualities	Diligent work	8/q15	They work very well and often notice things that anesthesiologists don't. For example, during a robotic-assisted surgery, when an emergency rollout occurred, they noticed the bleeding right away and immediately called the responsible anesthesiologist. Because they were able to respond immediately, the surgery was completed without causing any major problems.
			7/q52*	The PAN can administer anesthesia, immediately call for help if any issues come up, and also participate in education. Our PAN is polished.
		Heightened motivation	2/q109	They are more diligent than a poor doctor. For example, they are eager to write papers, present their work at conferences, and so on.
		Superior communication skills arising from mature personalities	6/q36	They have a good relationship with the anesthesiology residents. Perianesthesia nurses communicate better with them.
			17/q196	I recognize that our perianesthesia nurses are truly outstanding individuals.
		Academic thought processes	13/q45	They have the experience of doing research and writing a master's thesis. They take data, analyze it, summarize it, and then receive repeated supervision. This process enhances their ability to understand anesthesiology.
		Unique nursing-oriented cognition	10/q30	They are able to predict what is going to happen. In addition to being able to understand what is most important at all times during their work, they also take good care of their patients. They are attentive to the words and actions of the circulating nurse and give effective advice. At the same time, they should be aware of the patient's feelings. And they manage the patient's whole body. It is very impressive.
2	High-level tasks	Intraoperative anesthesia management	19/q28	PANs study for several years to understand the risks and importance of anesthesia before engaging in their work. Allowing PANs to administer anesthesia results in higher quality compared to having initial resident doctors do so.
			3/q19*	The PANs are capable of thinking for themselves. They don't rely on us too much. They can report, communicate, and consult about everything that's necessary. I've never had the experience of feeling scared because they did something on their own without consulting.
		Preoperative and postoperative tasks	11/q63	It is common for patients to share information with nurses that they may not feel comfortable telling doctors. Even when patients say they are fine during a doctor's examination, they may still express their concerns to a PAN when they visit. PANs possess unique skills as nurses, which is a priority in their role.

III. Anesthesiologists' expectations

	category	code	interview/ quote no.	quote summary
1	Expectations for PANs	Collaboration with anesthesiologists	5/q126	PANs thoroughly study perioperative care, including anesthesia management, enabling them to support anesthesiologists. They can also identify issues that anesthesiologists might overlook and provide valuable input. I hope that they will become professionals who can fulfill such roles.
		Enhanced inter-departmental cooperation	12/q237	Ward nurses sometimes lack understanding of surgery-related matters and may tell patients that pain is inevitable after surgery. The presence of nurses trained in postoperative care ensures proper management of nausea and pain. This contributes to the hospital's reputation for safety and high-quality care.
		Leadership and supervisory roles over other nurses	10/q80	Some circulating nurses may not have a strong interest in anesthesia. Anesthesiologists, due to job rotations, often find it challenging to engage with them consistently. PANs can serve as role models and leaders for these nurses.
		Dual perspectives (anesthesiologists and nurses)	10/q66*	Nurses' work is crucial. Meanwhile, anesthesiologists have various considerations in the operating room. However, there's no time to share these thoughts. PANs might best understand what both nurses and doctors are lacking. They are a unique profession. It would be beneficial to have individuals excelling in this role.
		Seamless perioperative patient care	20/q13	It is difficult to choose just one among preoperative, intraoperative, and postoperative care. The ability to handle a wide range of tasks is required for PANs. Preoperative evaluation is necessary for intraoperative management. Furthermore, those involved in intraoperative management should be aware of postoperative outcomes.
		Care skills	17/q176	Patients often feel more comfortable expressing their thoughts to nurses than to doctors. There may be surprisingly many such patients.
2	Benefits of the PAN system	Workload reduction	22/q7, 187	When PANs take charge of anesthesia management, anesthesiologists can avoid spending entire days in the operating room. This allows for efficient parallel execution of other tasks, such as preparing for preoperative clinics[...]. If PANs handle anesthesia preparation, our workload will be reduced.
		Anesthesiologists' engagement in advanced tasks	6/q98*	Well-trained PANs can conduct proper preoperative evaluations. Preoperative assessments are currently a burden on anesthesiologists. Reducing this burden can allow anesthesiologists to shift their resources to managing critically ill patients.
		Anesthesia care improvement	20/q18	Prehabilitation is becoming increasingly important. Depending on the patient's condition, a preparation process starting about a month before surgery is established, followed by surgery and the promotion of postoperative functional recovery. From this perspective, the importance of care naturally grows.
3	Exemplary PANs	Interpersonal skills	4/q211	Cooperativeness is a desired quality. PANs should be able to effectively interact with both doctors and nurses.
		Excellent anesthesia assistance	4/q109	Ideally, we would like to have three PANs. One would be responsible for preoperative anesthesia clinic, another would handle anesthesia for less complex cases, and the third would support various other miscellaneous tasks.
		Enjoying the business	15/q76	PANs who enjoy their work are preferred.
		Eagerness to learn	2/q214	Ideal PANs always study and have the motivation to acquire new knowledge. They update their knowledge to understand anesthesiologists' thought processes.
		Understanding of the nurse's position ("wakimae")	2/q152	The relationship with anesthesiologists is the most important. If a PAN does something that an anesthesiologist dislikes, this system will not work well. A master-servant relationship is considered a safe style.
			21/q326*	Wakimae... that's what it's about, yes. You see, they're not anesthesiologists after all.
		Non-monetary motivations	9/q71	There is a concern that if PANs earn more than night-shift nurses, some people might become envious. Those motivated primarily by money are not desirable; PANs should possess a genuine motivation for anesthesia.

IV. Anesthesiologists' perspectives on PAN anesthesia

Category 1. Conflicting views on PAN anesthesia

	subcategory	code	interview/ quote no.	quote summary
1	PAN anesthesia "Support"	Fundamental understanding of PAN system as a delegated anesthesia system	13/q98	The expected duties include pre- and post-operative care, as well as acute pain service. PAN anesthesia in the operating room is already common practice. Building on this, I have various expectations for their future roles.
		Social factors contributing to the promotion of PAN anesthesia	19/q73	There were only about 10 positions for anesthesiologists, yet there are 15 or even 20 operating rooms. We're told to keep all operating rooms occupied with surgeries every day. What should we do with the remaining rooms? This is how perioperative nurses came into being.
			7/q80	In countries like the United States, there are "nurse anesthetists". The existence of such a term indicates that there's no issue with nurses administering anesthesia.
			3/q130	With advancements in medication, anesthesia has become much simpler and more straightforward to administer.
		Experience of no issues with PAN anesthesia	7/q29	In my mind, working with PANs is like working with capable interns or skilled residents. I believe I can entrust them with responsibilities.
		Impact of PAN anesthesia on enhancing anesthesiologists' expertise	6/q97*	Anesthesiologists can shift their focus to handling anesthesia for patients with severe conditions. Their role is expanding to intensive care, participation in Rapid Response Team, pain management in childbirth, and sedation procedures outside the operating room. This broadens the scope of anesthesiology beyond just the operating room. It expands the range of work for anesthesiologists. As members of the Rapid Response Team, anesthesiologists contribute to improving overall hospital safety.
			7/q129	As nurses' capabilities expand, anesthesiologists should diversify their skills, balancing critical incident management, epidural anesthesia, nerve blocks, and specialized procedures such as cesarean sections. This evolving landscape prompts a sense of urgency for anesthesiologists. However, it may prove beneficial in encouraging anesthesiologists to further specialize in their subspecialties in the future.
			17/q224*	The entry of nurses into the field will increase competition, potentially requiring anesthesiologists to increase their efforts. This resistance is understandable, as anesthesiologists may fear losing their place in the workplace, especially when balancing life events like childbirth or childcare.
2	PAN anesthesia "NOT support"			
	(Not actively in favor)	Request for Intraoperative Anesthesia Maintenance Only	3/q82*	I expect PANs to have basic anesthesia management skills. However, my preference is for them to focus on perioperative support. I hope PANs can provide more assistance in areas where anesthesiologists are currently able to do only the minimum, such as preoperative patient orientation.
		Legal compliance-only physicians can make dosage decisions for anesthetics.	17/q51	The law explicitly states "direct instruction". For instance, when a PAN thinks a vasopressor is needed, I always tell them to call me. I do not allow PANs to make decisions about additional administration of narcotics.
		Expectation for utilizing nursing qualifications and experience (PAN's unique speciality)	10/q50*	I believe there are tasks that only a PAN can effectively manage, occupying a position between the roles of nurses and anesthesiologists. It's not about tasks that anyone could do, but rather tasks that require the specific position of a PAN. For example, conducting study sessions for nurses.
	(Explicitly oppose)	Physician priority in case assignment	11/q93	A medical license is crucial. Even if it's an unmotivated junior resident, when someone needs to be in the operating room and we must do something better than leaving it unattended, I would choose a physician. If one wants to perform anesthesia, they should become a doctor first and then come.
		Explicitly oppose	14/q176*	I believe that dedicated anesthesiologists should administer anesthesia.
		Legal groundwork first	14/q86	Nurses are required to always confirm with a physician before performing any procedure. This is the official stance. Therefore, there is a possibility of delays in treatment. The legal constraints are significant. Conversely, if there were a clearly defined list of procedures that nurses are allowed to perform, I believe it would lead to a situation where certain tasks could be authorized for nurses to perform.
			11/q128*	I cannot place trust in individuals with self-proclaimed status or even institutional certification unless it is officially endorsed by the government. Such individuals lack the necessary qualifications. If complications were to arise, it would be unfortunate for the PANs involved. Additionally, I do not believe I could assume responsibility in such situations. PANs are akin to children who are great drivers but lack legal driver's licenses. In extreme circumstances where I am incapacitated or unable to function, I might consider allowing them to operate. However, I cannot sanction their routine administration of anesthesia.
			14/q73*	The practice of nurses being responsible for anesthesia appears to be in a legally ambiguous area. Additionally, there are safety-related concerns as well...
		Doubts about the safety of PAN anesthesia.	14/q87	If it is simply left to nurses to administer anesthesia, safety becomes a concern. It would be problematic if the system were casually changed to allow non-physicians to perform this task. In reality, there are experienced nurses who may be more reliable than inexperienced resident physicians, but not all nurses are like this. A system that institutionally relies on nurses cannot ensure safety.
		Criticism towards supporters	11/q163	Those who want nurses to administer anesthesia can go ahead and let them do it. But I don't think that's a good idea. PANs are not nationally certified. There's no job definition, and some might say any interpretation is fine. While there's some logic to that, it's dangerous.
3	The anesthesiologists' perspectives in substituting anesthesiologists with PANs			
	(Agree)	Agree	9/q206	It's not good to be overly concerned with job titles. Just introduce yourself as "I'm so-and-so from anesthesiology, and I administer anesthesia".
		No legal obstacles to PAN anesthesia	24/q99	With a physician's orders, nurses can do anything, even regular nurses, not just PANs. In extreme cases, as long as there are orders, they can even perform intubation, drain insertion, or central venous catheterization.
		Disadvantages of limiting the scope of work assigned to PANs	9/q13	Our facility has too many severe cases. If we say PANs can't manage ASA-PS3 patients, they would be left with no work to do.
		Need to replace unnecessary anesthesiologists with PANs	16/q140	PANs are cost-effective. They have good knowledge of anesthesia and can handle less complex cases. They work at the hospital daily. As more such individuals emerge, there will be less need for freelancers. Only anesthesiologists with high-level skills or various subspecialties will remain. Mediocre anesthesiologists will be eliminated. Anesthesiologists should be exposed to such competition.
			9/q75, q216, 217*	I want to gradually replace freelance anesthesiologists with nurses. I wish those unwilling to work would leave. They are motivated by money. If they want money, they should also take responsibility. My aim is to reduce positions for freelance anesthesiologists with low aspirations, who only want to administer anesthesia without contributing to education or the organization.
	(Disagree)	Disagree	1/q146	It's ideal for PANs to have knowledge and skills approaching those of physicians. However, I don't want them to become indistinguishable from doctors.
		Prioritizing legal compliance	17/q80	It's a fundamental principle that PANs should only act under direct orders from anesthesiologists. They should not operate under comprehensive orders.
		Concerns regarding the use of PANs for cost reduction/reduction of anesthesiologist positions	22/q18	The effectiveness of PANs doesn't justify reducing the number of anesthesiologists. Are PANs here to reduce the number of anesthesiologists? It's questionable.
			6/q156*	There's a concern that PANs might become mere "anesthesia machines." This would be unfortunate for them, as they are here not just to increase case numbers, but to provide more perioperative care and nursing.
		Differences between physicians and nurses	3/q57	It's good to fulfill their wishes to some extent. However, I can't support them if they want to do exactly what doctors do.
		Legal status	3/q67	There's no difference in teaching or having them perform anesthesia management. However, the issue of responsibility is problematic. Residents have medical licenses, but nurses don't.
			17/q208*	In medicine, the law is paramount. Personally, allowing nurses to perform medical procedures is... This stance won't shift in the future, all in the interest of patient safety. No matter how much things evolve, what's not allowed remains not allowed.
		Educational backgrounds	11/q206	There's a stark difference in the academic foundation, such as anatomy and physiology. This distinguishes them from physicians.
			12/q248*	There is indeed a difference in knowledge levels. While some PANs study diligently, the fundamental basis of what they've learned differs from that of physicians.
		Clinical proficiency	19/q52	PANs cannot engage in discussions about a patient's medical history on par with internists. While anesthesiologists can recommend necessary tests, it's difficult for PANs to independently request an ultrasound examination.
			21/q232*	Even if PANs possess knowledge, decision-making is a separate matter. Only doctors can truly elicit the decision-making aspect.
		Patient perceptions	15/q28	It would be good if they study and become capable of managing severe cases. However, this increases their responsibility. There's concern about whether patients would accept if complications, troubles, or incidents occur post-surgery when anesthesiologists haven't performed the anesthesia.
			4/q133*	Perioperative complications can occur without anyone being directly at fault. In current Japanese practice, if an incident happens, the nature of the work might be scrutinized. Actions performed by a doctor might be accepted. However, if a qualified nurse performs the same action and complications arise, patients might react aggressively, even if it's just consequentialism.
		Resistance and Disappointment towards Substituting PANs for Anesthesiologists	22/q114	Do some anesthesiologists truly believe that PANs can replace us? It seems some might actually think so, but this is difficult to comprehend. Is this really acceptable?

Category 2. Anesthesiologists' concerns in PAN anesthesia

	subcategory	code	interview/ quote no.	quote summary
1	Increasing daily workload	Assigning cases requires careful consideration	6/q50	Sedation outside the operating room is typically administered by a perianesthesia nurse working alongside an anesthesiology instructor. Despite the need for advanced skills, there are limited opportunities for anesthesiology residents to participate and gain experience. I find this concerning.
		Educational Burden	9/q60	There are numerous individuals requiring education, including not only PANs but also medical students, interns, and residents. There aren't sufficient anesthesiologists available to instruct them.
		Burden of Simultaneous Supervision	22/q292	To efficiently utilize multiple PANs in the operating room, simultaneous anesthesia is necessary. While there are managerial benefits, the burden on the supervising anesthesiologist is increasing.
2	Inadequate PANs	Underestimating Anesthesia / Lack of Apprehension	22/q170	I am concerned about PANs just focusing on the monitor, not recognizing that "something minor can be fatal" and assuming "nothing will happen anyway".
		Depend on manuals	10/q108	It is unfortunate PANs do not take advantage of their own abilities, saying things like, "That's not my job," or "The doctor told me to do it, so I'll make a manual to handle it," or "If that's what the doctor tells me to do, I won't do that job anymore.
		No responsibility as a nurse	12/q317	Now is the time for nurses to assume responsibility. It is regrettable that they are irresponsible as "the physician is responsible for medical care." They just wait for the doctor's orders, do nothing even if the patient is suffering unless the doctor tells them to, etc.
		Mistaking oneself as a doctor	21/q318	There may be Perianesthesia Nurses who mistakenly see themselves as doctors, thinking they are great and amazing because they can administer anesthesia.
		No guarantee that the best will always enter	18/q145	The PANs who are currently working with us are serious people who have carved out their own paths. Ordinary people would be hard-pressed to have that level of motivation.
		Difficult to make all PANs ideal through education alone	24/q81	It is impossible to elevate all PANs to a high level through education alone. Personal aptitude, character, and motivation also have a significant impact.
3	Medical litigation resulting from anesthesia accidents involving PANs	No legal backing	9/q103	It is a fact that Perianesthesia Nurses are administering anesthesia without legal backing.
		No legal knowledge	4/q133	I am not familiar with the laws related to liability in case of problems and other associated aspects.
		No legal precedent	19/q86	You do anesthesia with a PAN, an apparent anesthesia accident comes along, and then the case goes to court. Unless we see the case law, we don't know if there is any difference compared to similar accidents done with residents. I think this will happen in the future. Anesthesiologists are anxious. All anesthesiologists are afraid of the trial.
		Responsibility lies with the anesthesiologist	19/q76	Let's assume that the anesthesia incident happened because of the inadequacy of the Perianesthesia Nurse. I believe the responsibility should be shouldered by the anesthesiologist who was supervising the PAN.
		Being a nurse is a disadvantage in lawsuits	9/q114	In a medical lawsuit, the question may be asked, "Why were there nurses in the operating room instead of doctors in the first place?" In that situation, the fact that the nurse was a very studious nurse might not be taken into consideration. The world has not yet fully accepted nurses who can administer anesthesia.
4	Potential societal non-acceptance of PAN anesthesia	Regional differences in acceptance	22/q116	In a rural hospital where there are no anesthesiologists, the acceptance of nurse-administered anesthesia might be more plausible. Even though they are not anesthesiologists, they are individuals who have received proper anesthesia training. However, in urban areas where there is no shortage of anesthesiologists, the absence of an anesthesiologist in the operating room can become a factor contributing to issues or provide a reason for patients to blame us.
		Negative feelings of patients	12/q111	When something goes wrong, the fact that it was done by a nurse rather than a doctor could evoke negative feelings in the patient.
5	Risks of the diminishing value of anesthesiologists	Risk of making anesthesiologists unnecessary	15/q69	One of the causes of anxiety is the notion that the nurses are excellent and can manage anesthesia well, so there is no need for an anesthesiologist anymore.
		Risk of damage to value of anesthesiologist	3/q66	There is a potential for interns to perceive that administering anesthesia is a task that nurses can handle, and it's not a task for us physicians.

Category 3. Essentials for safe PAN anesthesia

	subcategory	code	interview/ quote no.	quote summary
1	Common thought process between PANs and anesthesiologists	Shared understanding through order sheets	2/q01	Initially, we created order sheets with detailed instructions for drug administration to prevent mistakes. We established rules that required PANs to contact us when vital signs during anesthesia went beyond specified ranges. I thought this would be sufficient to avoid problems.
		Shared thought processes	2/q02*	Being able to work while we sort of understand what each other is thinking, that's like, the ideal situation.
			12/q228	What I expect from PANs is the ability to carry out instructions given by anesthesiologists. Furthermore, I want them to recognize what we anesthesiologists consider a crisis and share that perception. It's beneficial when PANs can share this understanding based on their medical knowledge.
		PANs adapting to anesthesiologists' preferences	2/q197	Anesthesiologists have different preferences in their approach to anesthesia, such as prioritizing speed or stability. PANs need to understand and adapt to these individual preferences.
2	Establishment of PAN education systems by anesthesiologists	Close communication with PANs	7/q08	I believe it's crucial to boost the morale of each PAN and maintain frequent communication with them, tailoring our approach to each individual.
		Understanding the risks of anesthesia	5/q131*	Even experienced anesthesiologists find a small percentage of anesthesia cases challenging. It's crucial for PANs to recognize this complexity. Without this understanding, they might overestimate their ability to manage anesthesia independently. PANs need to experience and acknowledge the inherent anxiety in anesthesia practice. It's our responsibility to convey this reality to them.
		Education by anesthesiologists	5/q01	Before entrusting anesthesia to PANs, it's essential that they receive thorough education from anesthesiologists. Without this, we cannot entrust them with such tasks.
3	Limiting complexity and number of cases when supervising multiple nurses	Appropriate staffing numbers	18/q52*	Individuals' capabilities and areas of responsibility tend to become naturally apparent over time. We usually manage two people, but emergencies may require overseeing three.
		Appropriate case selection	19/q13	Specialists should handle complex anesthesia cases, while nurses can manage lower-risk procedures.
4	Reassigning inadequate PANs	Consideration of interpersonal compatibility	1/q81	If, after extensive discussions, a PAN's approach remains incompatible with mine, I'd prefer they work with a different anesthesiologist.
		Pre-employment screening	16/q118	We may need to consider the option of not hiring individuals who seem likely to engage in dangerous practices.
		Reassignment of inadequate PANs to other departments	17/q265, 266	Continually including underqualified individuals will lower the overall quality of the PAN team. For those unsuited to being PANs, I'd personally like to help identify their other strengths and reassign them to more appropriate positions.
		Adherence to boundaries	21/q163*	I have my own boundaries, and I expect PANs to respect them. It's problematic if a PAN can't adhere to those boundaries. If managing them becomes difficult, we can't entrust responsibilities to that PAN.

V. Anesthesiologists' perspectives on the PAN system

	category	code	interview/ quote no.	quote summary
1	Challenges in the operation of PANs	Enhancing the educational system and elucidating goals	7/q87	I think PANs should be allowed to perform procedures typically done by junior residents, such as arterial line insertion or central venous puncture. There are hands-on training sessions for regional anesthesia, right? In my opinion, if they've properly completed hands-on training, they should be allowed to perform nerve blocks as well.
		Determining the optimal PAN count	9/q58	The current challenge is determining what positions to prepare for PANs in five years. There's an idea to create employment opportunities not just in university hospitals but also in community hospitals. However, there's still no clear framework for how these community hospitals will integrate PANs into their systems. There's concern about continuing to train PANs without securing positions for them. It's better to first establish proper employment opportunities before increasing the number of trainees.
		Clarifying accreditation criteria	17/q99	Setting performance goals for PANs is a very difficult issue. Originally, the philosophy was that PANs would aim to be at the pinnacle among nurses in anesthesia-related fields.
			1/q266, 271*	I want a well-organized system. [...] I might be the only one, but I feel like I don't fully grasp the PAN system. It would be easier if there were clear boundaries set, like "How much work can I entrust to PANs?" The current system is unclear, and I don't know its limits. I'm just not sure...
		Refining human resource management	13/q59	There's an issue regarding whether PANs should be part of the Nursing Department or the Anesthesiology Department. Currently, they are under the Nursing Department, so their work schedules are managed by the Head Nurse. PANs might prefer to officially be part of the Anesthesiology Department. If they were to separate from the Nursing Department, their work arrangements and hours could potentially become more flexible.
		Addressing demotivating factors for PANs	14/q59	PANs work hard and invest a lot of time to acquire skills and knowledge. However, there are no financial benefits. In fact, it's becoming a disadvantage, which is troubling. We've been telling the hospital to properly value PANs, but it's not working out. Moreover, the nursing career ladder is also an issue. Even if PANs continue their current work, they can't become head nurses. Their salaries don't increase either. It might be good if there was a system for continuous recruitment of new staff, but currently, there isn't.
		Considering the potential decline in the proficiency of operating room nurses	11/q170	When I heard about PANs rushing to assist anesthesiologists in anesthesia emergencies, I thought it wasn't very good. What are the circulating nurses doing? They're the ones who should be able to handle such situations. It's as if the circulating nurses are being treated like a dermatologist called for a Code Blue, who is then told by the later-arriving emergency physician or anesthesiologist, "You can go now, you're in the way." Once the PAN system is complete, I thought that circulating nurses will lose their chance to grow unless they aim to become PANs.
2	Recommendations for the advancement of the PAN system	Clarifying the benefits of PANs	8/q55	Increasing what PANs can do will help maintain their motivation in the long term. Officially certifying their qualifications and increasing their pay would also be good. If they can perform more procedures or do more things independently than they can now, they might be able to maintain motivation even after working as a PAN for many years.
		Enhancing awareness and understanding of the system within society	12/q301	I'd like the system to be clearer. There are multiple systems for nurses involved in anesthesia. Even in meetings, when abbreviations come up, I sometimes don't even know what they're referring to. Most people probably don't understand things like the Training System for Nurses Pertaining to "Specified Medical Acts", certified nurses, nurse practitioners, whether these various types of nurses have master's degrees or not, and so on.
		Fostering the development of high-level anesthesiologists	21/q146	We need anesthesiologists with strong teaching abilities. It's very important that these anesthesiologists consistently showcase their comprehensive capabilities to PANs. These anesthesiologists can handle not just anesthesia, but also emergency care and intensive care. They have broad knowledge. We need anesthesiologists that PANs would recognize as undeniably superior. The presence of such anesthesiologists can keep in check PANs who might overestimate their ability to handle anesthesia independently.
		Implementing new systems	15/q53	PANs typically work from 9:00 AM to 5:00 PM. However, this can be problematic when anesthesiologists with childcare responsibilities need to leave in the evening. A more flexible system allowing PANs to work afternoon to night shifts would be beneficial. This could cover cases where anesthesiologists need to leave but the surgery is not severe and ongoing anesthesia could be managed by a PAN. If the system could be improved so that PANs could play an active role in such cases, I would be even more satisfied.
3	Participation of stakeholders	Japanese Society of Anesthesiologists	3/q173	With a system left to individual hospitals, PANs face difficult problems when they transfer. Trying to set rules between hospitals is likely to cause trouble. It would be easier for us to work if a higher authority, namely the Society of Anesthesiologists, dictated, "This is how it should be done."
		Japanese Society for Perianesthesia Nursing and Medicine	6/q153	I'd like to see qualification certification, even if it's at the academic society level. That would make it easier for hospitals to provide additional compensation.
		Nursing Association	6/q129	PANs are valuable to anesthesiologists. I'm happy to work with them. However, it's nurses who create the system for nurses. Unless nurses take the initiative, the system won't continue.
		The Japanese government	18/q136, 172	PANs should be licensed. It would be good if the license was valid at any hospital. [...] The biggest flaw in this system is the lack of licensing or public policy.
