# Supplementary material for: Variabilities and contentions in anesthesiologists’ perspectives on Japanese perianesthesia nurses: A qualitative study
Source: PLoS One. 2024 Dec 31;19(12):e0313158. doi: 10.1371/journal.pone.0313158 (PMC11687901; doi:10.1371/journal.pone.0313158)
Supplement: Attachment — Submitted filename: S4 Appendix SRQR.docx [file pone.0313158.s006.docx]

|  | **S1 Appendix: Standards for Reporting Qualitative Research (SRQR)*** |  |
| --- | --- | --- |
|  |  | **Page no(s).** |
| **Title and abstract** | |  |
|  | **Title** - Concise description of the nature and topic of the study Identifying the study as qualitative or indicating the approach (e.g., ethnography, grounded theory) or data collection methods (e.g., interview, focus group) is recommended  REVIEWER COMMENTS:  “Amid chaos: a qualitative study of anesthesiologists’ perspectives on Japanese peranesthetist nurses”. – It is not clear where is the place of “amid chaos” in the manuscript’s arguments. This expression is cited twice, in the title and on page 27, line 500.  Main results and discussion point out the perspective of anesthesiologists on Japanese perianesthetist nurses: a qualitative study, as an exploratory perspective. | 1 |
|  | **Abstract** - Summary of key elements of the study using the abstract format of the intended publication; typically includes background, purpose, methods, results, and conclusions  REVIEWER COMMENTS:  The abstract reflects the content of the manuscript and invites the reader to read it in its entirety, except for the conclusions.  It is recommended to review the writing of the conclusions to highlight the main knowledge generated from the research and that responds to the objective. The choice of the type of qualitative research suits the perspective of exploring meanings and experiences. Qualitative evidence was only possible to be elicited because the "Japanese anesthesiologists' perspectives on the shift of tasks to nurses" was preliminarily explored (inaugural examination). | 2,3 |
|  |  |  |
| **Introduction** | |  |
|  | **Problem formulation** - Description and significance of the problem/phenomenon studied; review of relevant theory and empirical work; problem statement  REVIEWER COMMENTS:  The formulation of the problem is clear, despite not contextualizing the legal framework that supports the work of Japanese advanced practice nurses in anesthesia, named Nurses Anesthesists. Globally, there is tension between the physician and nurse workforce, with the scope of nurses' roles expanding to compensate for shortages in the physician workforce. Many physicians around the world have resisted itis implementation. Therefore, advanced practice nursing requires legal regulation and strong clinical preparation of nurses. International Council of Nurses recognized that the identification and context of nurses working in anesthesia vary in different parts of the world. It is also recognized that the profession is dynamic with changes in education cation, regulation and practice of nursing as it seeks to respond to health needs and changes the provision of health services.  It recommends clarifying the legal and educational contexts to underpin the research problem, from the perspective of qualitative research. See that lines 84-89 belong to method section | 3,4 |
|  | **Purpose or research questio**n - Purpose of the study and specific objectives or questions  REVIEWER COMMENTS:  Adequate | 4,5 |
|  |  |  |
| **Methods** | |  |
|  | **Qualitative approach and research paradigm** - Qualitative approach (e.g., ethnography, grounded theory, case study, phenomenology, narrative research) and guiding theory if appropriate; identifying the research paradigm (e.g., postpositivist, constructivist/ interpretivist) is also recommended; rationale**  REVIEWER COMMENTS:  The qualitative research method is more than describing data collection and data analysis techniques. According to the checklist used to write the manuscript, the method section of the manuscript follow the sequence that begins with the Qualitative approach and research paradigm. In other words, the text needs to announce that it is an exploratory qualitative study {approach] based on the interpretation [paradigm] of the participants'narratives [method]. As there is no previously established theoretical lens for analysis and interpretation, this part of the text remains unrecorded.  It is suggested that the method presentation sequence be reviewed, as described in the Checklist | 7 |
|  | **Researcher characteristics and reflexivity** - Researchers’ characteristics that may influence the research, including personal attributes, qualifications/experience, relationship with participants, assumptions, and/or presuppositions; potential or actual interaction between researchers’ characteristics and the research questions, approach, methods, results, and/or transferability  REVIEWER COMMENTS:  Adequate | 6 |
|  | **Context** - Setting/site and salient contextual factors; rationale**  REVIEWER COMMENTS:  It is not written or unclear. Part of context is described in the participants and sampling topic | 5,6 |
|  | **Sampling strategy** - How and why research participants, documents, or events were selected; criteria for deciding when no further sampling was necessary (e.g., sampling saturation); rationale**  REVIEWER COMMENTS:  Adequate to meet the objective, but unclear regarding exclusion criteria and recruitment plan. It is necessary to separate the recruitment phase from the interview development phase. As the context (research scenario and its characteristics) was not presented, the reader has difficulty understanding why 16 hospitals (public, private, teaching, general and so on) were accessed to invite the 304 anesthetists. Part of the data collection time took place during the Covid 19 pandemic, how was the field research developed? What was the effect of this period, with its sanitary measures, on data collection? | 5,6 |
|  | **Ethical issues pertaining to human subjects** - Documentation of approval by an appropriate ethics review board and participant consent, or explanation for lack thereof; other confidentiality and data security issues  REVIEWER COMMENTS:  Adequate, but it is dispersal showed in different parts of the manuscript as highlighted below:  Line 124 – confidentiality  Line 125 - interviewee's consent.  Lines 126-130 – “Prior to each interview, explicit written consent forms were obtained. Participants were provided with a written explanation of the study's purpose, methods, and data handling procedures. They were also informed that their participation was voluntary and that they could withdraw from the study at any time without any consequences.  All participants provided written informed consent before the commencement of the interviews.”  Linha 142 - confidentiality  Lines 200-202 – Registration of IRB  It suggests to join all the information regarding ethical issues to protect participants confidentialities and data security issues relating to online interview, pandemic times and so forth. | 6,7,11 |
|  | **Data collection methods** - Types of data collected; details of data collection procedures including (as appropriate) start and stop dates of data collection and analysis, iterative process, triangulation of sources/methods, and modification of procedures in response to evolving study findings; rationale**  REVIEWER COMMENTS:  In the participants and sampling topic, line 96, it states that the in-depth interview was carried out; but, on line 123 of the data collection topic it says that it was an unstructured interview.  To maintain coherence and consistency, information from the data collection procedure must be reviewed. You need to inform how long the interview lasted and how many times the same participant was interviewed. Was a new interview necessary? Did the participant have access to the interview after it was transcribed? | 6-7 |
|  | **Data collection instruments and technologies** - Description of instruments (e.g., interview guides, questionnaires) and devices (e.g., audio recorders) used for data collection; if/how the instrument(s) changed over the course of the study  REVIEWER COMMENTS:  Line 123 reads that Data were gathered through unstructured interview in person or via video conferencing. There is a description of the procedures for face-to-face interviews, but not for videoconferencing. On which platform was it carried out, how long did it last, how was confidential information handled considering the insecurity of the virtual environment? | 6,7 |
|  | **Units of study** - Number and relevant characteristics of participants, documents, or events included in the study; level of participation (could be reported in results)  REVIEWER COMMENTS:  It is reported in the method section | 5,6 |
|  | **Data processing** - Methods for processing data prior to and during analysis, including transcription, data entry, data management and security, verification of data integrity, data coding, and anonymization/de-identification of excerpts  REVIEWER COMMENTS:  Partially clear. As there is a stage of data collection via videoconference, the security management of this data was not explained. After transcribing the recorded interviews, what was the final destination of the recordings? How was the data processed in MAXQDA (line 169) and for what purpose? | 7 |
|  | **Data analysis** - Process by which inferences, themes, etc., were identified and developed, including the researchers involved in data analysis; usually references a specific paradigm or approach; rationale**  REVIEWER COMMENTS:  Adequate and clear | 7-11 |
|  | **Techniques to enhance trustworthiness** - Techniques to enhance trustworthiness and credibility of data analysis (e.g., member checking, audit trail, triangulation); rationale**  REVIEWER COMMENTS:  Adequate and clear | 8-11 |
|  |  |  |
| **Results/findings** | |  |
|  | **Synthesis and interpretation** - Main findings (e.g., interpretations, inferences, and themes); might include development of a theory or model, or integration with prior research or theory  REVIEWER COMMENTS:  Categories 1, 2 and 3 are not supported by quotations. The absence of illustration weakens the movement of analysis from the perspective of transferability and use of this empirical knowledge as an evidence in qualitative systematic reviews, for example.  Despite the richness of qualitative data, it needs to associate the qualitative evidence (narrative of participants) to one of the categories. It is missing illustration for categories 1 to 3. Table 2 highlights 5 subcategories of category IV, where is the other 10 subcategories. Maybe the manuscript need to be revised and the data presented accordingly with the **S2 Appendix: Final analytical framework** | 11-24 |
|  | **Links to empirical data** - Evidence (e.g., quotes, field notes, text excerpts, photographs) to substantiate analytic findings  REVIEWER COMMENTS:  Adequate | 11-24 |
|  |  |  |
| **Discussion** | |  |
|  | **Integration with prior work, implications, transferability, and contribution(s) to the field -** Short summary of main findings; explanation of how findings and conclusions connect to, support, elaborate on, or challenge conclusions of earlier scholarship; discussion of scope of application/generalizability; identification of unique contribution(s) to scholarship in a discipline or field  REVIEWER COMMENTS:  The manuscript could expand the discussion to the context of advanced nursing practice, since the findings showed tensions and sensitive issues related to the acceptance of nurse anesthetists replacing some functions traditionally occupied by physician specialists.  **See some publication:** Aberese-Ako M, Agyepong IA, Gerrits T, Van Dijk H. 'I Used to Fight with Them but Now I Have Stopped!': Conflict and Doctor-Nurse-Anaesthetists' Motivation in Maternal and Neonatal Care Provision in a Specialist Referral Hospital. PLoS One. 2015 Aug 18;10(8):e0135129. doi: 10.1371/journal.pone.0135129.  Aimei Mao, Jialin Wang, Yuan Zhang, Pak Leng Cheong, Iat Kio Van & Hon  Lon Tam (2020) Male Nurses’ Dealing with Tensions and Conflicts with Patients and Physicians: A Theoretically Framed Analysis, Journal of Multidisciplinary Healthcare, , 1035-1045, DOI:10.2147/JMDH.S270113 | 24-27 |
|  | **Limitations** - Trustworthiness and limitations of findings  REVIEWER COMMENTS: Part of the limitation topic belongs to the conclusion; see statement on line 537-539. It is unclear why suggest a “comprehensive nationwide quantitative study is imperative to ascertain the collective views of anesthesiologists in Japan”. | 27-28 |
|  | Conclusion – See the comments to the abstract section |  |
| **Other** | |  |
|  | **Conflicts of interest** - Potential sources of influence or perceived influence on study conduct and conclusions; how these were managed  REVIEWER COMMENTS: It is declared | 29 |
|  | **Funding** - Sources of funding and other support; role of funders in data collection, interpretation, and reporting  REVIEWER COMMENTS:  It is declared | 29 |
|  |  |  |
|  | *The authors created the SRQR by searching the literature to identify guidelines, reporting standards, and critical appraisal criteria for qualitative research; reviewing the reference lists of retrieved sources; and contacting experts to gain feedback. The SRQR aims to improve the transparency of all aspects of qualitative research by providing clear standards for reporting qualitative research. |  |
|  |  |  |
|  | **The rationale should briefly discuss the justification for choosing that theory, approach, method, or technique rather than other options available, the assumptions and limitations implicit in those choices, and how those choices influence study conclusions and transferability. As appropriate, the rationale for several items might be discussed together. |  |
|  |  |  |
|  | **Reference:** |  |
|  | O'Brien BC, Harris IB, Beckman TJ, Reed DA, Cook DA. **Standards for reporting qualitative research: a synthesis of recommendations.** *Academic Medicine*, Vol. 89, No. 9 / Sept 2014  DOI: 10.1097/ACM.0000000000000388 |  |
|  |  |  |
|  |  |  |
