# Supplementary material for: Variabilities and contentions in anesthesiologists’ perspectives on Japanese perianesthesia nurses: A qualitative study
Source: PLoS One. 2024 Dec 31;19(12):e0313158. doi: 10.1371/journal.pone.0313158 (PMC11687901; doi:10.1371/journal.pone.0313158)
Supplement: Attachment — Submitted filename: Response to reviewers_21M3008Tamai.docx [file pone.0313158.s007.docx]

Responding to peer reviewer comments

Dear Dr. Stefano Turi,

We sincerely thank the editor and reviewers for their valuable feedback on our manuscript. We have carefully considered all points raised and have revised our manuscript accordingly. The changes are highlighted within the document for easy reference. We believe these revisions have significantly improved the quality of our work and hope they adequately address the concerns raised.

**Comments to the Author**

**1. Is the manuscript technically sound, and do the data support the conclusions?**

Reviewer #2: Partly

**Response to Reviewer #2 : 1. Is the manuscript technically sound, and do the data support the conclusions?**

In response to the reviewer's comments, we have revised the Conclusion section. The details of these revisions are provided in Response 2 to Comment 2: Abstract / Conclusion.

**5. Review Comments to the Author**

**Reviewer #1:** Too long article that can be shorter by modifying extra data. Ref can be improved and more updated and needs more clarification in conclusion part. It would be better if the authors try to explain about the usefulness of the study findings for international audiences.

**Response to Reviewer #1 : 5. Review Comments to the Author**

We sincerely thank you for your valuable feedback. We have addressed your concerns as follows:

1. Interview data: We have carefully selected only the most representative and crucial quotations. Supplementary data is provided in Appendix 5, allowing us to remove some previously included quotes from the same code. The remaining quotations are essential for enhancing the paper's persuasiveness.
2. Redundancy: We have eliminated unnecessary repetitions and verbose descriptions, particularly in the discussion section.
3. Conclusion: We have revised the conclusion as detailed in our Response 2 to Comment 2: Abstract / Conclusion.
4. International relevance: We added a paragraph discussing how physician-nurse relationships affect task shifting and how our study's findings suggest the need for strategies considering both universal and Japan-specific factors. This paragraph emphasizes that our findings provide valuable perspectives for healthcare policymakers and researchers both in Japan and internationally. We also added a concluding remark: "Moreover, Japan's experience could serve as a valuable lesson for other countries, particularly those where the legal status of nurses with new specialties is not clearly defined.
5. References: We have added several new references and expanded the discussion on advanced practice nursing.

The overall word count has increased compared to the previous version. However, we believe that by addressing your feedback, we have significantly improved the quality and clarity of the paper..

**Reviewer #2**: First, I congratulate the authors for choosing the topic of advanced nurse anesthetist practice, from the perspective of doctors and not nurses.

The study investigated the perception of anesthesiologists regarding the role of the nurse anesthetist in advancing practice in the Japanese healthcare system. The nurse anesthetist system was implemented in Japan in 2010, 14 years later, the authors sought to understand the perception based on the experience of anesthetists who play the role of supervisor of this advanced practice nurse. The abstract explains the content of the manuscript and encourages the reader to read the full text. The advanced practice nurse (APN) is a way of inserting this professional into health services, especially to cover the shortage of doctors in general specialties, such as anesthesiology. The problem is well founded, with current bibliographic references on the topic. The method is appropriate and responds to the objective of the study, by choosing the interview (in-depth) as a way of obtaining qualitative data. The participant eligibility criteria led to obtaining a representative sample of the set of qualified narratives from the professionals who volunteered to participate in the interviews. The ethical issues of the field research were presented with clarity and sufficient detail to understand the sensitive nature of the topic, while preserving confidentiality. The interview's trigger question – "Could you please outline the primary responsibilities of perianesthesia nurses in your hospital?" – elicited narratives that allowed an in-depth analysis. It also used a questionnaire with a set of open-ended questions. The analysis procedures were anchored in the framework method”, which are well described and encourage the reader to understand how the categories were established.

Despite this, there are some points in the manuscript that could be better presented to ensure the scientific criteria of qualitative studies.

**Response to Reviewer #2 : 5. Review Comments to the Author :**

We sincerely thank the reviewer for their thorough and insightful evaluation of our manuscript. We appreciate the positive feedback on our choice of topic, methodology, and overall presentation of the study. We appreciate your insights on areas for improvement in our qualitative research. We are committed to carefully considering and responding to all your specific suggestions, including those aligned with SRQR guidelines, to strengthen our manuscript.

**Comment 1 :Title**

“Amid chaos: a qualitative study of anesthesiologists’ perspectives on Japanese peranesthetist nurses”. – It is not clear where is the place of “amid chaos” in the manuscript’s arguments. This expression is cited twice, in the title and on page 27, line 500.

Main results and discussion point out the perspective of anesthesiologists on Japanese perianesthetist nurses: a qualitative study, as an exploratory perspective.

**Response 1 : Title**

Thank you for your comment on the title. We chose the phrase "amid chaos" based on our observation that qualitative research papers sometimes use evocative expressions in their titles to symbolically represent the phenomenon under study. However, we appreciate your feedback and agree that a more direct title better represents our study's content and methodology. We have revised the title to "Variabilities and contentions in anesthesiologists' perspectives on Japanese perianesthesia nurses: a qualitative study." We believe this title accurately reflects our research focus and approach.

**Comment 2 :Abstract / Conclusion**

The abstract reflects the content of the manuscript and invites the reader to read it in its entirety, except for the conclusions. It is recommended to review the writing of the conclusions to highlight the main knowledge generated from the research and that responds to the objective. The choice of the type of qualitative research suits the perspective of exploring meanings and experiences. Qualitative evidence was only possible to be elicited because the "Japanese anesthesiologists' perspectives on the shift of tasks to nurses" was preliminarily explored (inaugural examination).

**Response 2 : Abstract / Conclusion**

We appreciate your feedback on our conclusion. We have revised both the main text and abstract conclusions to address your comments:

1. We clarified the study's purpose in the introduction.
2. The main conclusion now emphasizes:

- The diverse perspectives of Japanese anesthesiologists on PANs and the system.
- Significant variations in views on PANs' roles among institutions and individuals.

3. We explicitly stated the appropriateness of the qualitative approach.
4. We highlighted the significance of our findings in relation to task-shifting to nurses, emphasizing potential barriers identified.

Abstract Conclusion

This qualitative study is the first to reveal that Japanese anesthesiologists hold diverse perspectives on PANs and the system. The approach was well-suited for exploring diverse perspectives, showing significant differences among institutions and individuals. Our data provided crucial insights, including findings that suggest potential barriers to task shifting of anesthesia duties to PANs.

Main text Conclusion

This study is the first to reveal that Japanese anesthesiologists hold diverse perspectives on PANs and the system. The qualitative approach was well-suited for exploring their diverse perspectives. In particular, it was shown that perspectives on the role of PANs vary significantly among institutions and individuals, with some critical perspectives. The need for task-shifting to nurses has emerged as a strategic imperative to improve healthcare services and address rising medical costs. Our data provided crucial insights, including findings that suggest potential barriers to the task shifting of anesthesia duties to PANs.

**Comment 3 : Introduction**

The formulation of the problem is clear, despite not contextualizing the legal framework that supports the work of Japanese advanced practice nurses in anesthesia, named Nurses Anesthesists. Globally, there is tension between the physician and nurse workforce, with the scope of nurses' roles expanding to compensate for shortages in the physician workforce. Many physicians around the world have resisted itis implementation. Therefore, advanced practice nursing requires legal regulation and strong clinical preparation of nurses. International Council of Nurses recognized that the identification and context of nurses working in anesthesia vary in different parts of the world. It is also recognized that the profession is dynamic with changes in education cation, regulation and practice of nursing as it seeks to respond to health needs and changes the provision of health services.←査読者の解説は、

It recommends clarifying the legal and educational contexts to underpin the research problem, from the perspective of qualitative research. See that lines 84-89 belong to method section

**Response 3 : Introduction**

We sincerely appreciate the reviewer's comprehensive overview of the global context surrounding advanced practice nurses.

In response, we have made the following revisions:

1. we have added a brief explanation of the varied roles of nurse anesthetists worldwide and clarified the absence of equivalent positions in Japan.

Lines - in the revised manuscript "Nurse anesthetists provide anesthesia care in numerous countries. Their responsibilities, tasks, and roles vary significantly worldwide due to diverse historical backgrounds and healthcare systems [6].In Japan, there are no recognized positions equivalent to nurse anesthetists or anesthesia assistants."

2. We moved the following sentences to the "Context" subsection in the Methods:

Lines 68-70 in the original manuscript :"The nature of their duties varies based on the requirements of each facility, including pre-operative outpatient care, intra-operative anesthesia management, post-operative pain management, extra-operative sedation, and pain-free delivery assistance [8,9]."

3. To improve clarity, we concisely restructured the description of the importance of cooperation between physicians and nurses with new specialties.
4. We incorporated the following content into the "Qualitative approach and research paradigm" subsection in the Methods:

Lines 84-89 in the original manuscript: " This exploratory study was conducted to provide an in-depth analysis of anesthesiologists' perspectives and sentiments toward PANs. The qualitative research method focuses on individual experiences and emotions, providing a deeper insight into the current situation [16–18]. We believe this study can clarify some factors involved in developing the PAN system in Japan."

5. We have summarized the legal and educational background in the "Context" subsection of the Methods.

**Comment 4 :** **Qualitative approach and research paradigm**

The qualitative research method is more than describing data collection and data analysis techniques. According to the checklist used to write the manuscript, the method section of the manuscript follow the sequence that begins with the Qualitative approach and research paradigm. In other words, the text needs to announce that it is an exploratory qualitative study {approach] based on the interpretation [paradigm] of the participants'narratives [method]. As there is no previously established theoretical lens for analysis and interpretation, this part of the text remains unrecorded.

It is suggested that the method.

**Response 4 :** **Qualitative approach and research paradigm**

We appreciate the valuable feedback regarding the structure and content of our Methods section. In response to the reviewer's comments, we have made the following changes:

1. We have rearranged the structure of the Methods section to align with the SRQR guidelines.
2. We have added a new subsection titled "Qualitative approach and research paradigm".
3. To better articulate our research approach and clarify our study design, we have included the following sentence.

Line - in the revised manuscript : "In this study, we adopted an exploratory qualitative approach to analyze in detail the perspectives of anesthesiologists, using data from participants' narratives.

**Comment 5 : Context**

It is not written or unclear. Part of context is described in the participants and sampling topic.

**Response 5 : Context**

Thank you for your valuable input. We have created a new paragraph for "Context." We moved some of the descriptions from the "Participants and Sampling" section and added sentences related to the context. The additional content is as follows:

1. The number, geographical distribution, and implementation status of PAN training programs in Japan
2. Types of hospitals where PANs are employed
3. PANs' collaboration with anesthesiologists of diverse experiences and backgrounds
4. PANs' job responsibilities; discretion at each employing facility
5. Legal framework for PANs in Japan
6. Lack of unified standards or laws regarding PAN certification, education, and scope of practice

**Comment 6 : Sampling strategy**

Adequate to meet the objective, but unclear regarding exclusion criteria and recruitment plan. It is necessary to separate the recruitment phase from the interview development phase. As the context (research scenario and its characteristics) was not presented, the reader has difficulty understanding why 16 hospitals (public, private, teaching, general and so on) were accessed to invite the 304 anesthetists. Part of the data collection time took place during the Covid 19 pandemic, how was the field research developed? What was the effect of this period, with its sanitary measures, on data collection?

**Response 6 : Sampling strategy**

We appreciate the reviewer's detailed observations regarding our sampling strategy. In response to these valuable suggestions, we have made the following revisions:

1. We clarified the exclusion criteria and recruitment plan.
2. We described the recruitment phase and the interview development phase separately.
3. We clarified the process of accessing 16 hospitals and including all 304 anesthesiologists. We added details on how these hospitals were identified, the types of hospitals included, and the process of contacting and surveying anesthesiologists.
4. We add the sentence ; Of the 24 participants, 12 were from main hospitals and six from affiliated hospitals. The remaining participants included five from university hospitals without PAN programs and one from a general hospital.
5. The impact of the pandemic on our research process was addressed in the subsection "Ethical issues pertaining to human subjects" due to its direct relevance to participant consent, confidentiality, and data security protocols.

**Comment 7 : Ethical issues pertaining to human subjects**

Adequate, but it is dispersal showed in different parts of the manuscript as highlighted below:

Line 124 – confidentiality

Line 125 - interviewee's consent.

Lines 126-130 – “Prior to each interview, explicit written consent forms were obtained. Participants were provided with a written explanation of the study's purpose, methods, and data handling procedures. They were also informed that their participation was voluntary and that they could withdraw from the study at any time without any consequences.  All participants provided written informed consent before the commencement of the interviews.”

Line 142 - confidentiality

Lines 200-202 – Registration of IRB

It suggests to join all the information regarding ethical issues to protect participants confidentialities and data security issues relating to online interview, pandemic times and so forth.

**Response 7 : Ethical issues pertaining to human subjects**

We sincerely appreciate the reviewer's thorough examination of our manuscript's ethical considerations. In response to these insightful suggestions, we have made the following comprehensive revisions.

Ethical considerations that were previously dispersed throughout the manuscript were consolidated into a single section. This section is structured in three paragraphs: ethical review, participant consent, and other aspects of confidentiality and data security. Information about the impact of the pandemic was also included here. The destination of the recorded data, as pointed out in S13, was also addressed in this section.

**Comment 8 : Data collection methods, instruments, and technologies**

In the participants and sampling topic, line 96, it states that the in-depth interview was carried out; but, on line 123 of the data collection topic it says that it was an unstructured interview.

To maintain coherence and consistency, information from the data collection procedure must be reviewed. You need to inform how long the interview lasted and how many times the same participant was interviewed. Was a new interview necessary? Did the participant have access to the interview after it was transcribed?

**Comment 9 : Data collection instruments and technologies**

Line 123 reads that Data were gathered through unstructured interview in person or via video conferencing. There is a description of the procedures for face-to-face interviews, but not for videoconferencing. On which platform was it carried out, how long did it last, how was confidential information handled considering the insecurity of the virtual environment?

**Response 8,9 : Data collection**

We appreciate the reviewers' careful attention to the consistency and clarity of our data collection procedures. In response to these valuable comments, we have made the following comprehensive revisions:

1. We modified the subheading from "Data collection" to "Data collection methods, instruments, and technologies" to better align with the SRQR guidelines.

2. We clarified our use of terminology regarding interviews. Initially, we understood "unstructured interview" as "please speak freely" and "in-depth interview" as "please listen carefully for an extended period." After receiving feedback, we realized these ambiguous terms might compromise clarity. Therefore, we retained the term "unstructured interview" to express our approach of beginning with the question "How do PANs function in your hospital?" and allowing participants to speak freely. We removed the ambiguous term "in-depth interview" and instead explicitly stated that we spent considerable time listening to participants' narratives.

3. We added and reorganized information about the data collection procedure, including:

- The interview period (January 2022 to May 2023)

- The number of face-to-face vs. online interviews (17 out of 24 were online)

- The platform used for online interviews (Zoom)

- The recording methods for both face-to-face and online interviews

- Handling of confidential information in online interviews: We ensured both the participant and interviewer were in private locations and utilized Zoom's encryption features to maintain data security.

4. We specified the median interview duration (55 minutes, range: 37-96 minutes) and clarified that each participant was interviewed once, with no follow-up interviews conducted.

5. We added information about participants' right to review their interview transcripts, noting that none chose to exercise this option.

**Comment 10 : Data processing**

partially clear. As there is a stage of data collection via videoconference, the security management of this data was not explained. After transcribing the recorded interviews, what was the final destination of the recordings? How was the data processed in MAXQDA (line 169) and for what purpose?

**Response 10 : Data processing**

We appreciate the reviewer's comments. We have addressed these concerns as follows:

1. Security management of videoconference data:

We have added information in the "Ethical issues pertaining to human subjects" section regarding measures taken to ensure privacy during online interviews.

2. Final destination of recordings:

The "Ethical issues pertaining to human subjects" section now includes details on the storage and final disposal of recorded data, stating that recordings will be permanently deleted upon study completion.

3. We have added information about the use of a secure video conferencing platform (Zoom).
4. Data processing in MAXQDA:

We have added a new paragraph explaining the purpose and methods of data processing using MAXQDA. This includes details on how we used the software to systematically code and analyze our data, ensuring comprehensive coverage and preventing data omission.

**Comment 11 : Results/findings**

Categories 1, 2 and 3 are not supported by quotations. The absence of illustration weakens the movement of analysis from the perspective of transferability and use of this empirical knowledge as an evidence in qualitative systematic reviews, for example.

Despite the richness of qualitative data, it needs to associate the qualitative evidence (narrative of participants) to one of the categories. It is missing illustration for categories 1 to 3. Table 2 highlights 5 subcategories of category IV, where is the other 10 subcategories. Maybe the manuscript need to be revised and the data presented accordingly with the **S2 Appendix: Final analytical framework**

**Response 11 : Results/findings**

We appreciate the reviewer's comments regarding the presentation of our results. In our previously submitted manuscript, we described the highest level as 5 categories, with 15 subcategories below them, each containing multiple codes. However, in the complex Category IV, data within the subcategories were further classified into multiple lower-level subcategories, each with multiple codes. Table 2 in the manuscript was an example of one lower-level subcategory and its associated codes. Our explanation using the term "subcategory" in this table was insufficient. We have now revised the highest level to 5 "Themes," each containing 15 "Categories."

Based on this, we have made the following revisions:

1. We have created a new comprehensive table in the S5 appendix that clearly illustrates the hierarchy of the 5 themes and their structure, showing the codes belonging to each theme and category, along with data summaries and citation numbers.

2. Appendix 5 maintains consistency with the case-code matrix presented in Figure 1. We acknowledge that S2 Appendix provides an overview of each theme and keywords that could signify relationships between the themes.

3. We added representative narratives translated from interview excerpts to all former categories I-III (now Themes I-III).

4. We have reorganized the complex hierarchical structure of Theme IV. We have clarified the structure of Theme IV, namely the existence of three categories: 1. Conflicting views on PAN anesthesia, 2. Anesthesiologists' concerns in PAN anesthesia, 3. Essentials for safe PAN anesthesia, both in the main text and in Appendix 5. We have specified that each category has three, five, and four subcategories respectively, and that two of the three subcategories included in Category 1 are divided into two groups according to differences or conflicts in views. Table 2, which is one of the subcategories belonging to Category 2, continues to be included in the main text.

5. We have updated the terminology in Figure 1 and Appendices S2 and S3 to reflect these changes.

We believe these revisions provide a clear and comprehensive presentation of our results, addressing the reviewer's concerns about the structure and illustration of our data analysis.

**Comment 12 : Discussion**

The manuscript could expand the discussion to the context of advanced nursing practice, since the findings showed tensions and sensitive issues related to the acceptance of nurse anesthetists replacing some functions traditionally occupied by physician specialists.

**See some publication:** Aberese-Ako M, Agyepong IA, Gerrits T, Van Dijk H. 'I Used to Fight with Them but Now I Have Stopped!': Conflict and Doctor-Nurse-Anaesthetists' Motivation in Maternal and Neonatal Care Provision in a Specialist Referral Hospital. PLoS One. 2015 Aug 18;10(8):e0135129. doi: 10.1371/journal.pone.0135129.

Aimei Mao, Jialin Wang, Yuan Zhang, Pak Leng Cheong, Iat Kio Van & Hon

Lon Tam (2020) Male Nurses’ Dealing with Tensions and Conflicts with Patients and Physicians: A Theoretically Framed Analysis, Journal of Multidisciplinary Healthcare, , 1035-1045, DOI:10.2147/JMDH.S270113

**Response 12 : Discussion**

Thank you for your valuable suggestion to expand the discussion in the context of advanced nursing practice. We have made significant revisions to address this point, broadening our discussion to encompass a wider context and referencing the literature you introduced.

1. The revised discussion begins with a comprehensive overview of the study's main findings. We have synthesized key points from our original manuscript to create a new introductory paragraph that succinctly captures the diverse perspectives of Japanese anesthesiologists on PANs.
2. Using the study on male nurses' perceptions [29] recommended by the reviewer, we have added a paragraph discussing the gender ratio of PANs in Japan and its potential impact on "*wakimae*" and physician-nurse dynamics, specifically how the higher proportion of male PANs might influence hierarchical relationships and interprofessional collaboration.
3. We have removed the redundant summary of results.
4. We have maintained the paragraph that expands on the discussion of patient perceptions of nurses in Japan, highlighting the challenges this presents for the PAN system.
5. We have addressed how diverse perspectives on PAN roles can be a barrier to implementing advanced nursing practice.
6. Citing Chinese research and the Ghana study [35] recommended by the reviewer, we have discussed how role ambiguity can lead to various issues, including nurse burnout, conflicts between doctors and nurses, and a decline in care quality.
7. We have also emphasized the importance of incorporating both anesthesiologists' and PANs' opinions in role clarification processes.
8. We have mentioned that the PAN system in Japan was initiated without legal frameworks, leading to diverse and sometimes conflicting views among anesthesiologists. We have discussed the importance of legal frameworks and social dialogue for successful task shifting. As presented in "**Response to Reviewer #1, Comment 5:**", this section also develops the discussion within an international framework.

Based on the above, we were able to comprehensively discuss the challenges and possibilities of implementing advanced nursing practice, including expanding the discussion to an international perspective.

**Comment 13 : Limitations**

Part of the limitation topic belongs to the conclusion; see statement on line 537-539. It is unclear why suggest a “comprehensive nationwide quantitative study is imperative to ascertain the collective views of anesthesiologists in Japan”.

**Response 13: Limitations**

We sincerely appreciate the reviewer's comments. In response, we have revised our limitations section. Thanks to the reviewer's feedback, we now understand that the content in lines 537-539 (the view that anesthesiologists need to differentiate themselves by enhancing their capabilities may be influenced by participants affiliated with educational institutions) does not represent a limitation. Similarly, we now also understand that our proposal for a nationwide quantitative study was not sufficiently justified within the context of our qualitative research. Consequently, we have removed these statements.

Moreover, we have focused on the limited participation of less experienced anesthesiologists and have revised the limitations section accordingly. We initially described anesthesiologists who had completed their initial clinical training in Japan and were undergoing further training to become anesthesiology specialists as "held fellowship positions." However, to improve clarity for an international readership, we decided to change this term to "senior resident." Consequently, we have updated the terminology throughout the manuscript. Specifically, we now describe the breakdown of participants as "three were senior residents, four were specialists, and 17 served as supervisors.

Your feedback has significantly improved our manuscript's quality and clarity. We sincerely thank the editor and reviewers for their valuable insights. We have carefully considered each comment and incorporated all suggested improvements. We hope that these revisions adequately address the concerns raised and meet the high standards of PLoS ONE. We look forward to your further comments and remain open to any additional suggestions for improvement. Thank you for your time and expertise in reviewing our work.

Sincerely,

MIKIKO TAMAI
