# Supplementary material for: Variabilities and contentions in anesthesiologists’ perspectives on Japanese perianesthesia nurses: A qualitative study
Source: PLoS One. 2024 Dec 31;19(12):e0313158. doi: 10.1371/journal.pone.0313158 (PMC11687901; doi:10.1371/journal.pone.0313158)
Supplement: Attachment — Submitted filename: Response to reviewers_R2_21M3008Tamai.docx [file pone.0313158.s008.docx]

Responding to peer reviewer comments

Dear Dr. Stefano Turi,

We sincerely thank you for considering the revised version of our manuscript. We have carefully considered your additional comment that we "did not completely follow the indications of Reviewer 1 'Too long article that can be shorter by modifying extra data'".

In response to your suggestion, we have made efforts to modify excess data to shorten the article and present some parts differently to make our work more easily interpretable. We believe these revisions have significantly improved the quality of our work and hope they adequately address the concerns raised.

Below, we detail the specific revisions we have made:

**1. Overall Condensation and Improved Readability**

We have shortened the text in 20 places throughout the manuscript to enhance readability. Specifically:

- Methods section:
  - Context: 2 places
  - Participants and sampling: 5 places
  - Ethical issues pertaining to human subjects: 5 places
  - Data collection methods, instruments, and technologies: 5 places
- Discussion section: 3 places

**2. Changed Presentation of Participant Characteristics**

In the Participants and sampling part of the Methods section, we have presented participant characteristics (tenure, position, and gender distribution) in Table 1. This allowed us to visually represent information that was previously explained in text, thereby reducing the amount of text.

**3. Visualization of Data Analysis Process**

We have added Fig 1. "Data analysis process" to the Data analysis section and revised the accompanying text accordingly. This visualization enabled us to explain the analysis process more concisely.

**4. Concise Interview Quotations in Results**

We have added the following statement to the Results section: "To enhance readability and conciseness of the research, interview quotations have been carefully condensed to eliminate conversational redundancies without altering the original meaning."

Following this principle, we have carefully condensed 24 interview quotations. This process of condensing allowed us to significantly reduce the overall length of the Results section while preserving the original meaning of the quotes.

The specific list of condensed interview quotations is as follows:

| Theme | Case/ Quote No. | Original Text | Condensed Text |
| --- | --- | --- | --- |
| I | 9/q87 | In community hospitals and others, they're severely understaffed, and on top of that, with the COVID situation, a lot of anesthesiologists were taken up by the ICU. Well, not so much 'taken up,' but more like they had no choice but to go there. So then, who's left to handle anesthesia in the operating rooms? | In community hospitals and others, they're severely understaffed, with COVID-19, many anesthesiologists were taken up by the ICU. -they had no choice but to go there. Who's left to handle anesthesia in the operating rooms? |
|  | 16/q163 | I think the Japan Society of Anesthesiologists or some organization like that needs to handle the registration of perianesthesia nurses, you know? We gotta make sure they are placed where anesthesiologists are present because if they start working alone with surgeons, it'll be a huge problem. | The Japan Society of Anesthesiologists or similar organization should handle perianesthesia nurse registration. They must be placed with anesthesiologists as working alone with surgeons would be a huge problem. |
| II | 3/q19 | The PANs are capable of thinking independently, and they don't rely on us too much. They make sure to confirm everything that is necessary and consistently keep us in the loop by reporting, communicating, and consulting with us. I haven't had any experiences so far where I felt scared that they went ahead and did something without touching base with us first. | The PANs think independently, and don't overly rely on us. They　confirm necessary things and keep us informed through reporting, communication, and consultation. I’ve never experienced feeling scared that they executed something without consulting us. |
|  | 7/q152 | The perianesthesia nurse can administer anesthesia and immediately call for help if any issues come up, and also participate in education. Our perianesthesia nurse is just ideal. | The perianesthesia nurse can administer anesthesia, call for help if issues arise, and participate in education. Our perianesthesia nurse is ideal. |
| III | 10/q66 | Nurses' work is probably really important, and we have various things to think about in the operating room, you know? We don't even have time for mutual conferences, do we? So, we need to, how should I say this, understand a bit more about what each other is thinking while working... What's lacking, well, what the doctors here are lacking, what the nurses are lacking - I think the perianesthesia nurses probably understand this best. They're, how should I put it, a unique profession. I think it would be great if there were quite a few people who could really excel in that role. | Nurses' work is probably really important, and we have various things to think about in the operating room. Without time for mutual conferences, we need to understand more about what each other is thinking while working... Perianesthesia nurses likely best understand what doctors and nurses are lacking. They're a unique profession. I think it would be great to have quite a few people excelling in that role. |
|  | 6/q98 | You know, in a sense, once PANs have received proper training, they can handle quite a lot of preoperative evaluations. Our work involves quite a lot of these things, actually. So, to sum it up, we want to gradually shift anesthesiologists' resources towards more critically ill patients and core anesthesia-related tasks. | Once properly trained, PANs can handle many preoperative evaluations. Our work involves quite a lot of these things, actually. So, to sum it up, we want to gradually shift anesthesiologists' resources towards more critically ill patients and core anesthesia-related tasks. |
|  | 21/q326 | It's *Wakimae*... that's what it's about, yes. You see, they're not anesthesiologists after all. | It's *Wakimae*... that's what it's about, they're not anesthesiologists after all. |
| IV | 6/q97 | So, first off, anesthesiologists can sort of shift their focus to handling anesthesia for patients with severe conditions. And more, we're talking about diving into intensive care, jumping into action with the rapid response team, making deliveries less painful, and dealing with sedation stuff outside the operating room. It's a way to broaden the anesthesiologist gig, you know? And it's not just about the surgery room. We're talking about taking care of things all over the hospital, like being on point with the Rapid Response Team and contributing to improving overall hospital safety. | Anesthesiologists can shift their focus to patients with severe conditions. And more, they can dive into intensive care, join rapid response team, manage labor pain, and dealing with sedation stuff outside the operating room. It's a way to broaden the anesthesiologist role. It’s not just about the surgery room. We're taking care of things all over the hospital, being on point with the Rapid Response Team and contributing to improving overall hospital safety. |
|  | 17/q224 | With nurses entering the field, there will be increased competition. I believe this implies that anesthesiologists may need to exert more effort than they currently do. I can comprehend how anesthesiologists might emotionally resist, fearing that when life events like childbirth or childcare align, they might find no place to return to in the workplace. | Nurses entering the field will be increased competition. I believe this may require anesthesiologists to exert more effort. I can comprehend their emotional resistance, fearing when life events like childbirth or childcare align, they might find no place to return to in the workplace. |
|  | 3/q82 | I'd want PANs to have at least a minimum level of anesthesia management skills. However, my preferred setup is for them to focus on perioperative support. I wish PANs could offer a bit more assistance with preoperative patient orientation and other tasks that anesthesiologists can only do minimally. | I want PANs to have at least minimum anesthesia management skills, but prefer they focus on perioperative support. I wish PANs could offer a bit more assistance with preoperative patient orientation and tasks that anesthesiologists can only do minimally. |
|  | 10/q50 | I feel like there's stuff that only a PAN can handle, you know, things that fall right in between a nurse and an anesthesiologist. | I feel like there's stuff that only a PAN can handle, things that fall right in between a nurse and an anesthesiologist. |
|  | 14/q73,q176 | I feel like having a nurse handle anesthesia is a bit of a legal gray area. And, you know, safety-wise, too...(omission) I reckon dedicated anesthesiologists should be the ones giving anesthesia. | I feel like having a nurse handle anesthesia is a bit of a legal gray area. And, safety-wise, too...(omission) I reckon dedicated anesthesiologists should give anesthesia. |
|  | 11/q126 | I can't trust just anyone with a 'self-proclaimed,' or rather, an 'institutional certification holder' status unless it's officially endorsed by the government. It means they lack the qualifications, and I feel sorry for PAN if something goes wrong. I also feel I can't take responsibility. To put it in perspective, PAN is like a child who's a great driver but doesn't have a driver's license. So, in a real emergency where I'm unconscious or can't move, I might ask them to drive, but I can't let them routinely administer anesthesia." | I can't trust 'self-proclaimed' or 'institutional certification holders' without government endorsement. It means they lack the qualifications, and I feel sorry for PAN if something goes wrong. I can't take responsibility. PANs are like children who are great at driving but don't have licenses. In a real emergency where I'm unconscious or can't move, I might ask them to drive, but I can't let them routinely administer anesthesia. |
|  | 9/q75, q216, q217 | I, for one, want to get rid of more and more freelance anesthesiologists and replace them with nurses. I mean, I want people who don't work to leave (omission 1). They're just after money. I'm not saying they shouldn't make money, but the main point is if they want money, they should also take responsibility (omission 2). I aim to gradually phase out positions for freelance anesthesiologists with low aspirations, those who only want to do their own anesthesia, instead of mentoring younger staff at their own facilities and being involved in various operations within the organization | I want to replace more freelance anesthesiologists with nurses. I want people who don't work to leave (omission 1). They're just after money. I'm not saying they shouldn't make money, but if they want money, they should also take responsibility (omission 2). I aim to gradually phase out positions for freelance anesthesiologists with low aspirations, who only want to do their own anesthesia, not mentoring younger staff or being involved in various organizational operations. |
|  | 17/q208 | In medicine, everything revolves around the law. Personally, having a nurse carry out a medical procedure... This stance won't shift in the future, all in the interest of patient safety. Some things are just a no-go, regardless of how much things evolve. | In medicine, everything revolves around the law. Personally, having a nurse perform a medical procedure... This stance won't shift in the future, all for patient safety. Some things remain a no-go, regardless of evolution. |
|  | 12/q248 | The level of knowledge varies for sure. Some PANs put in a lot of effort into their studies, but the foundational aspects of what they've learned are distinct. | Knowledge levels vary for sure. Some PANs study hard, but the foundations of what they've learned are distinct. |
|  | 21/q252 | Even if PANs possess the knowledge, their ability to make decisions is a separate matter. I think only doctors can really pull off the decision-making part. | Even if PANs possess the knowledge, decision-making ability is a separate matter. I think only doctors can really handle the decision-making part. |
|  | 4/q133 | Even if it's not directly caused by someone's actions, there's still a chance that something might happen during the perioperative period. When that happens, in today's Japan, there's a possibility of scrutiny regarding the nature of the work if an incident occurs. If a doctor performed the act, well, it would be acceptable. However, if a nurse, even if qualified, did it, and something happened... Even if it was consequentialism, when that occurs, the patient's side might also become aggressive. | Even if it's not directly caused by someone's actions, there's a chance something might happen during the perioperative period. In JAPAN today, this may lead to scrutiny of work nature. If a doctor acted, it’s acceptable. However, if a nurse, even if qualified, did it, and something happened... Even if consequential, the patients might become aggressive. |
|  | 6/q156 | The PAN will just turn into another 'anesthesia machine.' It's a bummer for the PANs. They're willing to be here to handle more perioperative care and nursing, not just to bump up the case numbers. | The PAN will become just another 'anesthesia machine.' It's disappointing for them. They're here to handle more perioperative care and nursing, not just to increase the case numbers. |
|  | 2/q42 | Being able to work while we sort of understand what each other is thinking, that's like, the ideal situation. | Being able to work while we sort of understand what each other is thinking, the ideal situation. |
|  | 5/q131 | There are some aspects in anesthesia that are really tough to handle, maybe just a few percent, even after years of experience. I reckon if PANs don't recognize that, they might make the mistake of thinking, 'We can handle anesthesia on our own.' They need to experience the anxiety that comes with anesthesia. We also need to let them know that. | Some aspects of anesthesia remain tough to handle, maybe a few percent, even after years. If PANs don't recognize this, they might think, 'We can handle anesthesia alone.' They need to experience the anxiety that comes with anesthesia. We also need to let them know that. |
|  | 18/q92 | You'd expect the ability and areas where someone can take responsibility to kind of define themselves naturally. Normally, we're just keeping tabs on two people. But in a pinch, we might have to look after three. | You'd expect the ability and areas where someone can take responsibility to kind of define themselves naturally. Normally, we monitor two people. In a pinch, we might handle three. |
|  | 21/q163 | I've got my boundaries, and I want PAN to stick to those. If PAN crosses that line easily, I'm thinking, 'Uh-oh, I'm in a pickle.' If it's hard to manage, then I can't just leave it to that PAN. | I've got my boundaries, and I want PAN to stick to them. If PAN crosses them easily, I think, 'Uh-oh, I'm in a pickle.' If it's hard to manage, then I can't leave it to that PAN. |
|  | 1/q266,q271 | I want a well-organized system. (omission) Maybe it's just me, but I might not fully understand the PAN system. It would be easier if they clearly set boundaries like, 'How much work am I allowed to trust PAN to do?' The current system is unclear, and I'm uncertain about its limits. I don't know... | I want a well-organized system. (omission) Maybe it's just me, but I might not fully understand the PAN system. It would be easier if they clearly set boundaries on how much work to trust PAN with?' The current system is unclear, and I'm uncertain about its limits. I don't know... |

**5. Renumbering of Tables and Figures**

Due to the addition of the new Table 1 and Figure 1, we have renumbered the existing tables and figures as follows:

- Former Tables 1-3 → New Tables 2-4
- Former Figures 1-3 → New Figures 2-4

**6. Updated Reference**

We have thoroughly reviewed all references cited in the manuscript:

- No retracted papers were found among the citations.
- Updated the 9th reference from "Epub ahead of print" to its final publication details: 2024;39: 246–253.
- Revised access dates for all online articles to September 24, 2024, reflecting the most recent verification.

**7. Updated SRQR Guideline in Supporting Information**

We have updated the SRQR guideline (S4 Appendix) to accurately reflect the revised page numbers and content structure resulting from our manuscript modifications.

We believe these revisions have improved the conciseness and readability of the manuscript, addressing your comments. In particular, by modifying excess data and shortening the text, the overall manuscript has become more compact. Additionally, by effectively presenting some data in tables and figures, we have reduced the amount of text while maintaining clarity of information.

We sincerely thank Reviewer #2 for their favorable evaluation of our manuscript.

We appreciate their recognition of our efforts to incorporate previous recommendations and adhere to the SRQR checklist. Their insights have been invaluable in enhancing the quality of our research.

We sincerely thank the editor and reviewers for their valuable insights.

We believe these revisions have significantly improved the conciseness and readability of our manuscript, addressing your concerns while maintaining the scientific merit recognized by Reviewer #2. We hope that this revised version now meets the high standards of PLOS ONE and adequately addresses all the points raised during the review process."

Sincerely,

MIKIKO TAMAI
